# Low serum triglycerides related to delayed neurocognitive recovery in geriatric oral and maxillofacial surgery patients: A prospective cohort study

**DOI:** 10.1186/s12944-025-02819-9

**Published:** 2025-12-11

**Authors:** Jing Wang, Yanyong Cheng, Fan Wang, Chuanyu Qi, Yi Gao, Tiannan Chen, Jiayi Wang, Jinnan Xu, Ren Zhou, Yanan Jiang, Haoli Mao, Jia Yan

**Affiliations:** 1 Department of Anesthesiology, Shanghai Ninth People’s Hospital, Shanghai Jiao Tong University School of Medicine, No.639 Zhizaoju Road, Huangpu District, Shanghai, China; 2 Shanghai Jiao Tong University School of Medicine, No.227 Chongqingnan Road, Huangpu District, Shanghai, China

**Keywords:** General anaesthesia, Delayed neurocognitive recovery, Lipid metabolites, Oral and maxillofacial surgery, Geriatric patients

## Abstract

**Background:**

Geriatric patients undergoing oral and maxillofacial surgery are at high risk of delayed neurocognitive recovery (dNCR), yet reliable predictive tools remain unavailable.

**Methods:**

This prospective cohort study (July 2021–January 2025) enrolled patients aged ≥ 65 undergoing elective oral and maxillofacial surgery under general anaesthesia. Mini-Mental State Examination (MMSE) and Montreal Cognitive Assessment (MoCA) were assessed at baseline and postoperative 1, 3, 7, and 30 days. Serum lipidomics analysis via liquid chromatography-mass spectrometry was performed preoperatively and 24 h postoperatively. The predictive performance of lipid metabolites for dNCR was assessed using receiver operating characteristic curve analysis, with their independent association further evaluated by logistic regression.

**Results:**

Among 160 patients, 52 patients (32.5%) developed dNCR. Preoperatively, dNCR patients exhibited significantly lower serum triglyceride (TG), particularly TG(58:7/22:5) (OR = 0.014, 95% CI 0.002 to 0.109, adjusted *P* < 0.001) and TG(54:2/18:1) (OR = 0.051, 95% CI 0.010 to 0.252, adjusted *P* = 0.002), which demonstrated strong predictive performance (AUC = 0.86, sensitivity = 0.73, specificity = 0.85). Postoperatively, reduced levels of TG(58:7/22:5) (OR = 0.067, 95% CI 0.015 to 0.309, adjusted *P* = 0.003) and TG(54:2/18:1) (OR = 0.034, 95% CI 0.006 to 0.176, adjusted *P* < 0.001) persisted in dNCR patients at 24 h, retaining predictive value for dNCR (AUC = 0.82, sensitivity = 0.75, specificity = 0.78).

**Conclusions:**

Low serum TG(58:7/22:5) and TG(54:2/18:1) are promising biomarkers for early prediction of dNCR, supporting lipidomics-guided perioperative neurocognitive risk stratification.

## Introduction

With an aging population and increasing surgical indications, postoperative complications, particularly perioperative neurocognitive disorders (PND), in elderly patients have become critical healthcare priority [1, 2]. Among PND subtypes, delayed neurocognitive recovery (dNCR) manifests as newly acquired cognitive impairment persisting for up to 30 days postoperatively [3]. With reported incidence ranging from 25.8% to 34.5% in geriatric surgical populations, dNCR is associated with adverse outcomes, including prolonged hospitalization, functional decline and even increased mortality, highlighting the urgent need for early detection and intervention [4–7].

Patients undergoing oral and maxillofacial surgeries face an elevated risk of dNCR due to the anatomical complexity of the surgical site and substantial intraoperative hemodynamic fluctuations. Meta-analyses indicate that postoperative delirium (POD) rates in this population (21%–22%) exceed those of other elective non-cardiac surgeries (18%–19%), suggesting unique predisposition to neurocognitive dysfunction [8–11]. Despite this heightened risk, reliable predictive tools for dNCR remain unavailable in clinical practice.

Current approaches to predicting dNCR primarily rely on inflammatory or nutritional biomarkers, which were limited for delayed detection windows or cerebrospinal fluid sampling [8, 12–18]. Emerging evidence suggests that lipid metabolism may influence cognitive function through neuroinflammation, oxidative stress, and mitochondrial dysfunction [19]. Therefore, lipid metabolism disorders may promote neurodegenerative diseases, such as Alzheimer’s disease and Parkinson’s disease, through the above mechanisms. General anesthetics, being lipophilic, can interact with the blood-brain barrier (BBB), potentially exacerbating neurocognitive impairment [20]. Thus, abnormal lipid metabolism may induce measurable changes in lipid levels that predict postoperative neurocognitive decline, yet no studies have identified this relationship.

Prior studies in cardiac surgery patients have identified associations between altered lipid metabolites and dNCR [14, 21]. However, due to the different metabolic changes in various surgical patients, the results are not universally applicable. To address this gap, we conducted a prospective cohort study employing widely targeted lipidomics to characterize serum lipid profiles in patients undergoing elective oral and maxillofacial surgery to facilitate early risk stratification and formulate personalized perioperative cognitive protection strategies.

## Methods

### Ethics

This prospective cohort study was conducted in Shanghai Ninth People’s Hospital, Shanghai JiaoTong University School of Medicine, between July 2021 and January 2025. Ethical approval for this study (SH9H-2021-T120) was provided by the Ethical Committee of Shanghai Ninth People’s Hospital, Shanghai, China (Chairperson Prof Meng Luo) on 29 April 2021. A data analysis and statistical plan was written and filed with the Ethical Committee of Shanghai Ninth People’s Hospital before data was accessed. The study was registered with clinicaltrials.gov (NCT05105451) prior to patient enrollment. All assessments complied with the Declaration of Helsinki. Written informed consent was obtained before enrollment. This manuscript adheres to the applicable STROBE guidelines.

### Study design

Participants were patients aged 65 and older with an American Society of Anesthesiologists (ASA) level of I-III, scheduled for elective oral and maxillofacial surgeries under general anaesthesia. Exclusion criteria included history of mental or central nervous system diseases (epilepsy, Alzheimer’s disease, schizophrenia); history of preoperative psychotropic drug use; preoperative cognitive dysfunction; visual, auditory and language disorders; preoperative anxiety or depression; POD; history of perioperative rescue.

### Statistical power calculation

PASS 15.0 software (NCSS, LLC, Utah, US) was used for sample size calculation. According to the definition of dNCR, the diagnosis required a difference for 1 standard deviation (SD) between groups. Assuming a type I error level of 0.05, statistical power greater than 90% and a dropout rate of 10%, 25 cases for every group were required. Previous studies have shown that the incidence of dNCR is around 25%-35% [4–7]. Based on the incidence, the total sample should be at least more than 75 cases (25 cases in dNCR group and 50 cases in non-dNCR group) to meet the study requirements. To achieve superior statistical power and enhance the robustness of our findings, this study ultimately enrolled a total of 160 participants. This final sample size substantially exceeded the minimum requirement, ensuring a greater capacity to detect smaller effect sizes and strengthening the reliability of the study’s conclusions.

### Outcomes

The primary outcome was the occurrence of dNCR. According to the recommendations for the nomenclature of PND (2018) and Diagnostic and Statistical Manual of Mental Disorders 5 (DSM-5), dNCR is defined as a decline of at least 1 SD for neuropsychological test result [3, 22, 23]. In this study, we assessed dNCR by Mini-Mental State Examination (MMSE) and Montreal Cognitive Assessment (MoCA) at 1 day pre-surgery and at 1, 3, 7, 30 days post-surgery. DNCR was diagnosed if a decline of ≥ 1 SD (calculated by the baseline scores of all patients) in both MMSE and MoCA scores post-surgery was observed.

Medical history, laboratory examinations, psychological and cognitive evaluations using validated tools were recorded. Self-rating Depression Scale (SDS) and Self-rating Anxiety Scale (SAS) were used to exclude patients with preoperative anxiety or depression (standard scores of any scale greater than 50). At the same time, MoCA and MMSE were used to assess the baseline cognitive function, and the patients with preoperative cognitive dysfunction were excluded (preoperative MMSE score < 17 (illiterate), < 20 (elementary education), or < 24 (secondary education and above)). All assessments were conducted in a dedicated, quiet room by research staff who had received standardized training.

### Anaesthesia

All patients received general anaesthesia with tracheal intubation using a standardized perioperative care. Dorasetron (12.5 mg) and penehyclidine hydrochloride (0.5 mg) were routinely adopted as preoperative drugs. Anaesthesia induction included midazolam (0.01–0.04 mg kg^− 1^), sufentanil (0.2–0.3 µg kg^− 1^), propofol (0.8–1.5 mg kg^− 1^), rocuronium (0.6–0.8 mg kg^− 1^). Maintenance anesthetics contained sevoflurane (1%−2.5%), propofol (1–6 mg kg^− 1^ h^− 1^), remifentanil (0.05–1 µg^− 1^ kg^− 1^ min^− 1^), and were supplemented intermittently with rocuronium. Fluid therapy was administered using lactated or acetate Ringer’s solution and colloidal solution. Blood transfusion was considered if hemoglobin was less than 80 g L^− 1^. Throughout the surgeries, basic vital signs, bispectral index (BIS) and respiratory parameters were continuously monitored (volume-controlled ventilation, tidal volume: 6–8 ml/kg, oxygen concentration: 60%−80%, respiratory rate: 10–16 f min^− 1^, end-tidal carbon dioxide: 35–45 mmHg). Arterial blood gas analyses were performed every two hours. Postoperative analgesia was managed with pentazocine (30 mg).

### Sample collection

Arterial blood (5 mL) was collected before anaesthesia and at 24 hour post-surgery through an indwelling catheter placed in the radial artery. After stored at 25 °C for 15–30 min, samples were centrifuged (4 °C, 3000 rpm, 15 min). The supernatant obtained was frozen at −80 °C until analysis [24].

### Liquid chromatography–mass spectrometry

The serum sample and − 30 °C pre-cooled methanol (ThermoFisher) were mixed and fully vortexed, and then − 30 °C pre-cooled MTBE (Sigma-Aldrich) was added before a full vortex [25]. After that, ultra-pure water pre-cooled at 4 °C was added and vortexed together at 4 °C for 5 min at 2000 rpm. After centrifugation at 4 °C for 10 min at 12,000 g, 200 µL of the supernatant was transferred for vacuum drying. The dry samples were redissolved and vortexed for 5 min at 2000 rpm with 50 µL solution (acetonitrile (ThermoFisher): water = 95:5 (v/v)), and then centrifuged at 4 °C for 10 min at 17,000 g. At last, 2 µL of the supernatant was collected for liquid chromatography–mass spectrometry (LC-MS).

TSQ Altis mass spectrometer (Thermo Scientific) was used for metabolomics analysis. BEH Amide (1.8 μm, 100 × 2.1 mm) was selected as the chromatographic column. Mobile phase A was water: acetonitrile = 50:50 (v/v), and mobile phase B was water: acetonitrile = 5:95 (v/v) (both containing 10 mM ammonium acetate (ThermoFisher) + 0.2% ammonia water (Merck). At the column temperature of 45 °C, gradient elution was adopted with the flow rate of 0.30 mL/min and an injection volume of 2 µL. 

Ion detection was performed under the following source settings: sheath gas flow 40 Arb, auxiliary gas flow 10 Arb, sweep gas flow 1 Arb, ion transfer tube temperature 320°C, and vaporizer temperature 325 °C. The spray voltage was 3.5 and 2.8 kV for positive and negative polarity, respectively. The Q1 and Q3 resolutions were 0.7 and 1.2 Da, respectively. For the CID gas pressure, 1.5 mTorr was used.

### Metabolomics analysis

 Lipid molecules from each sample were measured through LC-MS and data was extracted by Xcalibur 4.0 (Thermo Fisher Scientific, USA). Lipids with > 50% loss were eliminated and missing values were filled in with a minimum value of 1/5. Lipid molecules were analyzed by MetaboAnalyst 6.0 (https://www.metaboanalyst.ca). The data was first normalized by Z-score standardization. Then orthogonal partial least squares discriminant analysis (OPLS-DA) was applied to screen differential metabolites. Variable importance in projection (VIP) of OPLS-DA was computed to describe the overall contribution of each metabolite to the discrepancies. Meanwhile, Student t-test was performed and fold-change (FC) was calculated to judge the significance of differential metabolites by SPSS 26 (SPSS, Chicago, IL, United States). False discovery rate (FDR) was calculated by Benjamini-Hochberg procedure to adjust *P*-values. Due to the sample size and biochemical characteristics of lipid metabolites in this study, differential metabolites were included for further analysis if VIP > 2.0 with FDR < 0.05, and FC > 1.20 or < 0.83.

### Statistical analysis

SPSS 26.0 was used to analyze the data. Normally distributed continuous data was expressed as mean (SD) and compared by Student t-test. Continuous data that was not normally distributed was reported as median (interquartile range) and compared by Wilcoxon test. Count data was expressed as frequency (percentage), and chi-square test was used. *P*-value < 0.05 (two-sided) indicated statistical significance.

For metabolites selected for further investigation, the receiver operating characteristic (ROC) curve analysis was conducted and the area under the curve (AUC) was calculated using Prism 9 (GraphPad Software, CA, USA). The optimal cutoff point, sensitivity and specificity were determined based on the highest Youden index. In addition, an exploratory analysis of the predictive power of pairwise metabolite combinations was performed to enhance the predictive potential. ROC curve analysis was used to evaluate the discrimination of metabolite combinations. Logistic regression was then used to analyze the association between lipid metabolites and the occurrence of dNCR. *P*-values from ROC curve analyses (testing AUC against 0.5) and logistic regression were adjusted for multiple comparisons using the Bonferroni method.

## Results

### Demographic and clinical characteristics

**Fig. 1:**
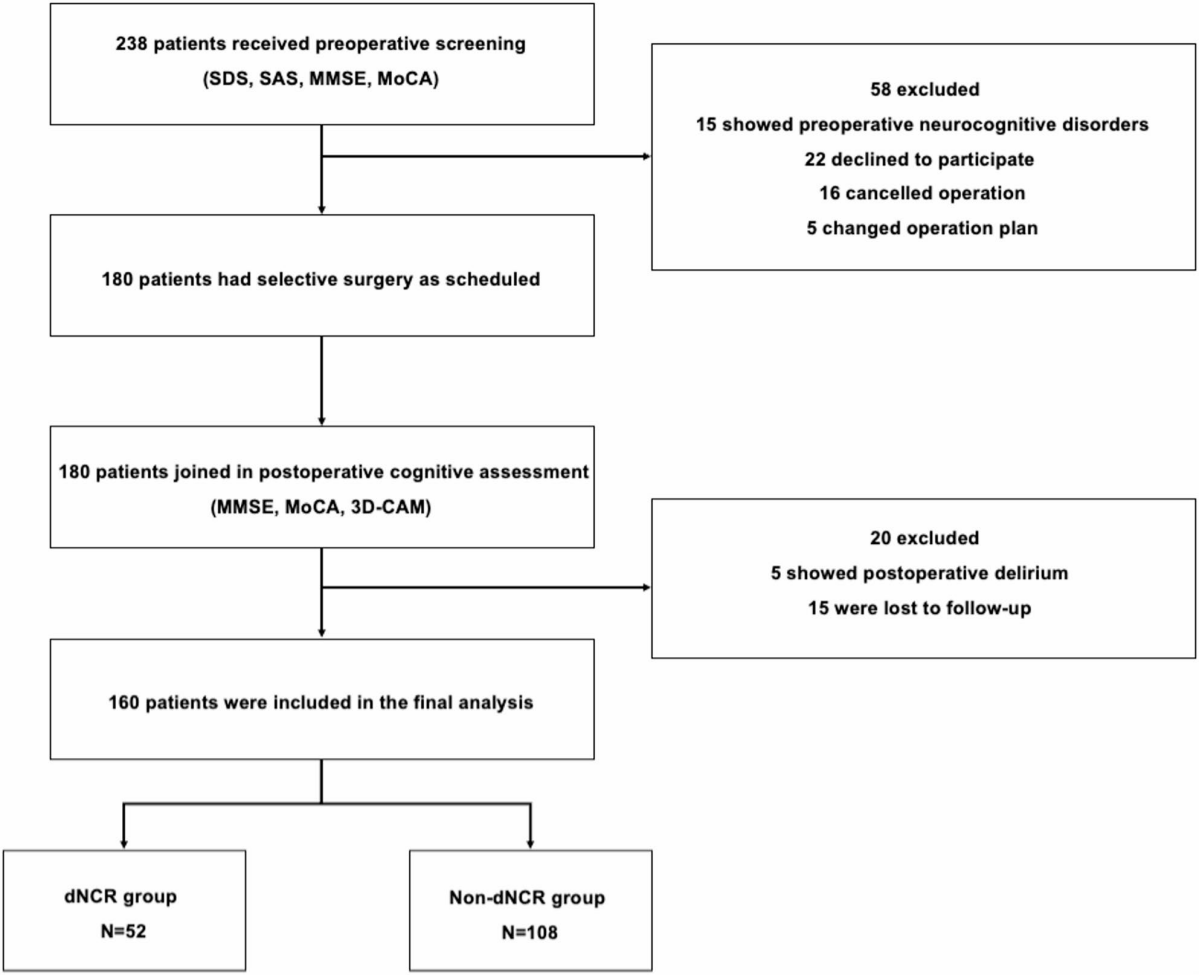
Flow chart. 238 patients were initially recruited. 58 patients were excluded due to preoperative cognitive impairment, withdrawal of informed consent, surgery cancellation, or anaesthetic method alteration. 180 patients completed baseline cognitive assessments and blood sample collection. 20 patients were excluded because of postoperative delirium (POD), or loss to follow-up. Consequently, 160 patients completed all cognitive evaluations (postoperative days 1, 3, 7, and 30) and lipid metabolomics analysis. Among 160 patients, 52 patients (32.5%) developed dNCR. SAS = Self-Rating Anxiety Scale; SDS = Self-Rating Depression Scale; MMSE = Mini-Mental State Examination; MoCA = Montreal Cognitive Assessment; 3D-CAM = 3-minute Diagnostic Interview for Confusion Assessment Method; dNCR = Delayed neurocognitive recovery

 238 patients scheduled for elective oral and maxillofacial surgery under general anaesthesia between July 2021 and January 2025 were initially recruited. 58 patients were excluded due to preoperative cognitive impairment, withdrawal of informed consent, surgery cancellation, or anaesthetic method alteration. 180 patients completed both baseline cognitive assessments and blood sample collection. Postoperatively, 20 additional patients were excluded because of POD, or loss to follow-up. Consequently, 160 patients completed all cognitive evaluations (postoperative days 1, 3, 7, and 30) and lipid metabolomics analysis (Fig. 1). No serious complication occurred during the perioperative period.

**Fig. 2:**
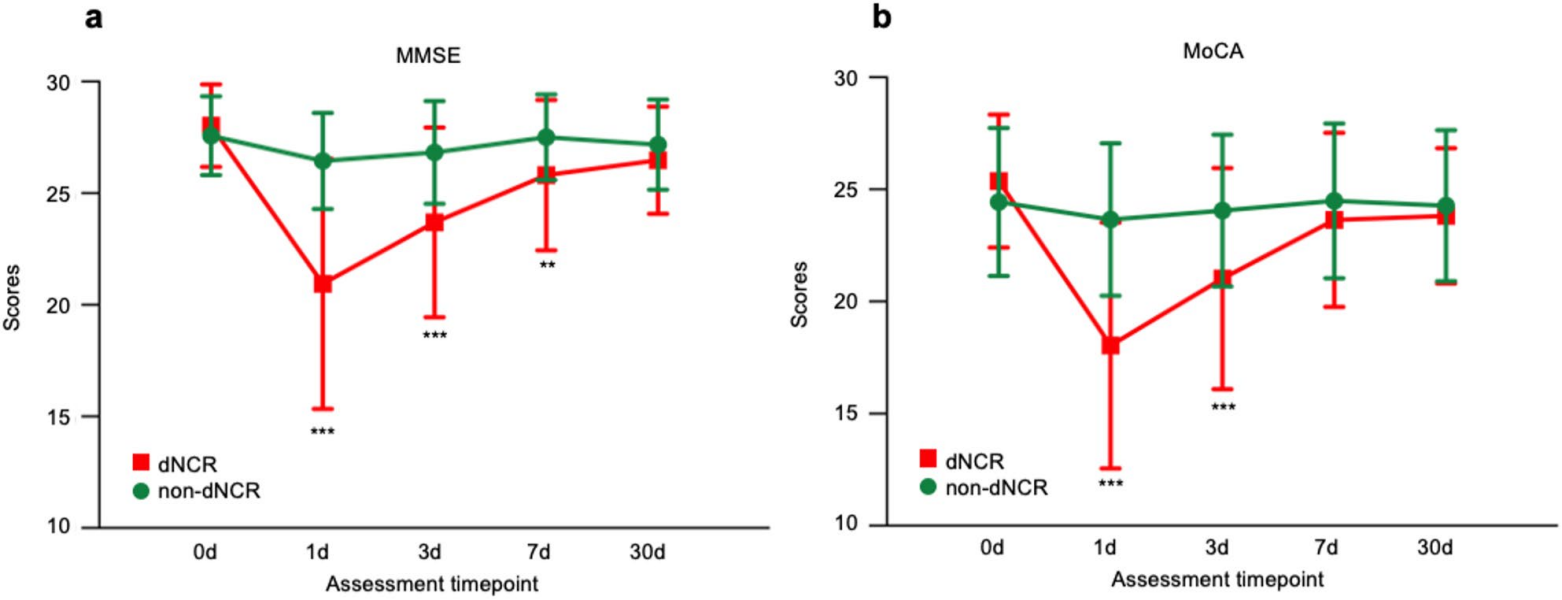
Preoperative and postoperative MMSE and MoCA scores. **a** Line chart of perioperative MMSE scores in dNCR and non-dNCR groups. **b** Line chart of perioperative MoCA scores in dNCR and non-dNCR groups. *** indicates *P*-value < 0.001. ** indicates *P*-value < 0.01. MMSE = Mini-Mental State Examination; MoCA = Montreal Cognitive Assessment; dNCR = Delayed neurocognitive recovery

Demographic and preoperative assessment data was detailed in Table 1. The mean and SD of preoperative baseline scores were 27.7 (1.8) for MMSE and 24.7 (3.2) for MoCA among all participants. Patients demonstrating a decline of ≥ 2 points in MMSE and ≥ 4 points in MoCA at any postoperative evaluation timepoint were then classified into the dNCR group (*n* = 52). Cognitive function demonstrated a marked acute decline on the first postoperative day. Following this initial deterioration, most patients transitioned into a rapid recovery phase, with the dNCR incidence rate dropping to 5.6% by postoperative day 30 from 26.3% initially. Notably, approximately 3.1% of patients maintained persistent cognitive impairment through day 30 without recovery, indicating the existence of a chronic protracted subtype. The overall cumulative incidence of 32.5% establishes dNCR as a prevalent complication among geriatric oral and maxillofacial surgery patients (Fig. 2).

**Table 1:** Demographic and clinical characteristics

	Non-dNCR group	DNCR group	*P*-value
Case number	108	52	
Sex			
Male	55(50.9%)	35(67.3%)	0.05
Female	53(49.1%)	17(32.7%)	
Age; years	71.9(5.5)	71.0(5.3)	0.29
Education level			
College	7(6.5%)	6(11.5%)	0.14
Junior college	11(10.2%)	1(1.9%)	
High school	24(22.2%)	10(19.2%)	
Junior high school	39(36.1%)	19(36.5%)	
Primary school	26(24.1%)	13(25.0%)	
Illiterate	1(0.9%)	3(5.8%)	
Height; cm	163.8(8.0)	165.8(8.3)	0.16
Weight; kg	62.1(11.7)	64.4(9.9)	0.23
ASA			
I	1(0.9%)	1(1.9%)	0.88
II	92(85.2%)	44(84.6%)	
III	15(13.9%)	7(13.5%)	
SAS score	30.5(4.8)	28.6(3.6)	**0.01**
SDS score	32.7(5.3)	30.8(3.8)	**0.01**
MMSE-pre score	27.6(1.8)	28.0(1.9)	0.15
MoCA-pre score	24.4(3.3)	25.4(3.0)	0.09
RBC (×10^12^/L)	4.4(0.6)	4.3(0.6)	0.35
Hemoglobin (g/L)	133.1(18.3)	131.2(17.1)	0.54
WBC(×10^9^/L)	12.4(66.7)	6.4(2.5)	0.51
Platelet (×10^9^/L)	212.4(58.0)	198.7(57.0)	0.16
Total albumin(g/L)	69.6(6.0)	67.6(6.7)	0.05
Creatinine(µmol/L)	68.1(17.3)	71.5(15.6)	0.24
PT(s)	11.3(0.7)	11.2(0.8)	0.34
APTT(s)	26.2(2.4)	25.6(2.5)	0.14
Blood glucose(mmol/L)	6.1(1.9)	5.7(1.4)	0.24
ALT(U/L)	22.4(17.1)	20.7(9.3)	0.49
AST(U/L)	24.3(11.8)	22.6(7.3)	0.32
HR-lowest; bpm.min^− 1^	66.0(9.8)	64.6(6.6)	0.29
MAP-lowest; mmHg	82.6(10.2)	80.4(9.2)	0.18
Electrocardiogram			
Normal	107(99.1%)	51(98.1%)	0.61
Premature pulse	1(0.9%)	1(1.9%)	
Bleeding volume; ml	163.1(210.4)	265.4(242.4)	**0.01**
Anesthesia duration; min	173.4(159.2)	252.0(189.3)	**0.01**

Values are mean (SD), median (IQR) or number (proportion) for categorical measures

*dNCR *Delayed neurocognitive recovery*, SAS *Self-Rating Anxiety Scale, *SDS *Self-Rating Depression Scale, *MMSE *Mini-Mental State Examination, *MoCA *Montreal Cognitive Assessment, *RBC *Red blood cell, *WBC *White blood cell, *PT *Prothrombin time, *APTT *Activated partial thromboplastin time, *ALT *Alanine aminotransferase, *AST *Aspartate aminotransferase, *HR *Heart rate, *MAP *Mean arterial pressure, *BIS *Bispectral index

Except for SAS, SDS, anaesthesia duration and blood loss (*P* < 0.05), no statistically significant difference was found in other demographic and clinical characteristic data between dNCR and non-dNCR patients (*P* > 0.05) (Table 1).

### Preoperative and postoperative differential lipid metabolites

**Fig. 3:**
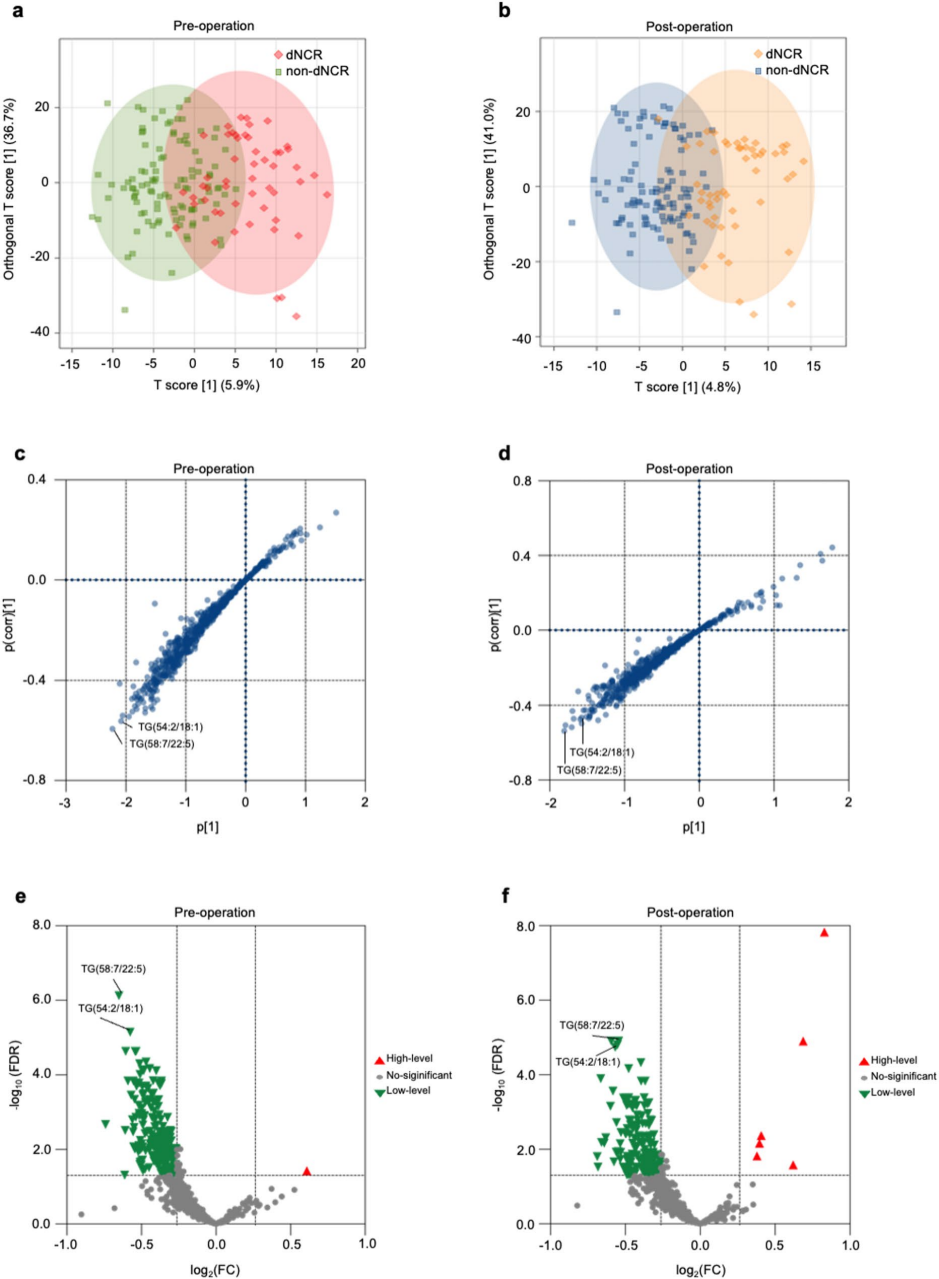
Preoperative and postoperative OPLS-DA and volcano plots. **a** Score plot of OPLS-DA of preoperative serum lipid metabolites from dNCR and non-dNCR groups. The OPLS-DA score plot demonstrated clear separation trend between the preoperative serum of dNCR and non-dNCR patients. **b** Score plot of OPLS-DA of postoperative serum lipid metabolites from dNCR and non-dNCR groups. The OPLS-DA score plot showed remarkable separation between the postoperative serum of dNCR and non-dNCR patients. **c** S-plot of OPLS-DA analysis of preoperative serum lipid metabolites. The plot showed that TG(58:7/22:5) and TG(54:2/18:1) had significant variation between dNCR and non-dNCR groups. **d** S-plot of OPLS-DA analysis of postoperative serum lipid metabolites. The plot showed that metabolites such as TG(58:7/22:5) had significant variation between dNCR and non-dNCR groups. **e** Volcano plot of preoperative different lipid metabolites. TG(58:7/22:5) and TG(54:2/18:1) were significantly lower in the preoperative serum of dNCR patients. **f** Volcano plot of postoperative different lipid metabolites. TG(58:7/22:5) and TG(54:2/18:1) were significantly lower in the postoperative serum of dNCR patients. dNCR = Delayed neurocognitive recovery; FDR = False discovery rate; FC = Fold-change; TG = triglyceride

LC-MS analysis identified 646 lipid metabolites. OPLS-DA score plot of preoperative and postoperative serum samples exhibited discernible spatial separation trend between dNCR and non-dNCR patients (Fig. 3a, b). S-plots highlighted significant differences in serum lipid metabolites between dNCR and non-dNCR patients, both before and after surgery (Fig. 3c, d). Differential lipid metabolites between the two groups were identified through multiple comparisons with FDR adjustment and FC criteria (Fig. 3e, f).

From the differentially abundant lipids, we identified several strong candidate metabolites preoperatively, most notably TG(58:7/22:5) (VIP = 2.45, FC = 0.64, *FDR* < 0.001) and TG(54:2/18:1) (VIP = 2.32, FC = 0.67, *FDR* < 0.001), which demonstrated the most substantial alterations. A complete list of top candidates was shown in Table 2.

**Table 2:** Preoperative and postoperative differential lipid metabolites between dNCR and non-dNCR patients

Lipid metabolomics	VIP	FC	Relative Intensity, mean(SD)		*FDR*
			Non-dNCR	dNCR	
Preoperative					
TG(58:7/22:5)	2.45	0.64	1.01(0.38)	0.64(0.23)	7.49E-07
TG(54:2/18:1)	2.32	0.67	1.00(0.37)	0.67(0.23)	7.14E-06
TG(54:3/18:1)	2.24	0.70	0.94(0.35)	0.66(0.23)	5.05E-05
TG(58:8/22:5)	2.23	0.66	0.96(0.39)	0.63(0.26)	2.34E-05
TG(52:2/18:1)	2.18	0.76	0.88(0.27)	0.67(0.21)	7.79E-05
TG(58:7/18:1)	2.16	0.69	0.94(0.35)	0.64(0.24)	2.34E-05
TG(52:3/16:1)	2.12	0.72	0.89(0.35)	0.65(0.23)	1.97E-04
TG(52:3/18:1)	2.12	0.78	0.83(0.24)	0.65(0.21)	1.46E-04
FA(18:1)	2.11	0.75	0.87(0.26)	0.65(0.27)	1.44E-04
TG(52:2/16:0)	2.08	0.78	0.87(0.26)	0.67(0.21)	1.69E-04
Postoperative					
PE(O-16:0/18:1)	2.97	1.78	1.08(0.53)	1.92(0.94)	1.47E-08
CL(72:3/18:2)	2.78	1.61	0.88(0.39)	1.42(0.81)	1.24E-05
TG(58:7/22:5)	2.46	0.66	0.97(0.36)	0.64(0.30)	1.24E-05
TG(56:5/20:4)	2.36	0.68	0.98(0.34)	0.67(0.31)	1.58E-05
TG(56:4/20:4)	2.31	0.67	1.08(0.41)	0.73(0.31)	1.84E-05
TG(54:3/18:1)	2.27	0.69	0.92(0.33)	0.63(0.24)	1.24E-05
TG(52:2/18:1)	2.19	0.76	0.90(0.24)	0.68(0.25)	4.77E-05
TG(58:7/18:1)	2.17	0.72	0.94(0.37)	0.68(0.33)	6.12E-04
TG(58:8/22:5)	2.16	0.67	0.95(0.42)	0.63(0.35)	2.74E-04
TG(56:5/18:0)	2.15	0.72	0.93(0.31)	0.67(0.28)	6.89E-05

*dNCR *Delayed neurocognitive recovery*, SD *Standard deviation*, FC* Fold-change, *VIP *Variable importance in projection, *TG *Triglyceride, *FA *Fatty acid, *PE *Phosphatidylethanolamine, *CL *Cardiolipin

*P*-values were adjusted by false discovery rate (FDR) and presented by scientific notation

### Low serum TG(58:7/22:5) and TG(54:2/18:1) as predictive biomarkers for dNCR

**Fig. 4:**
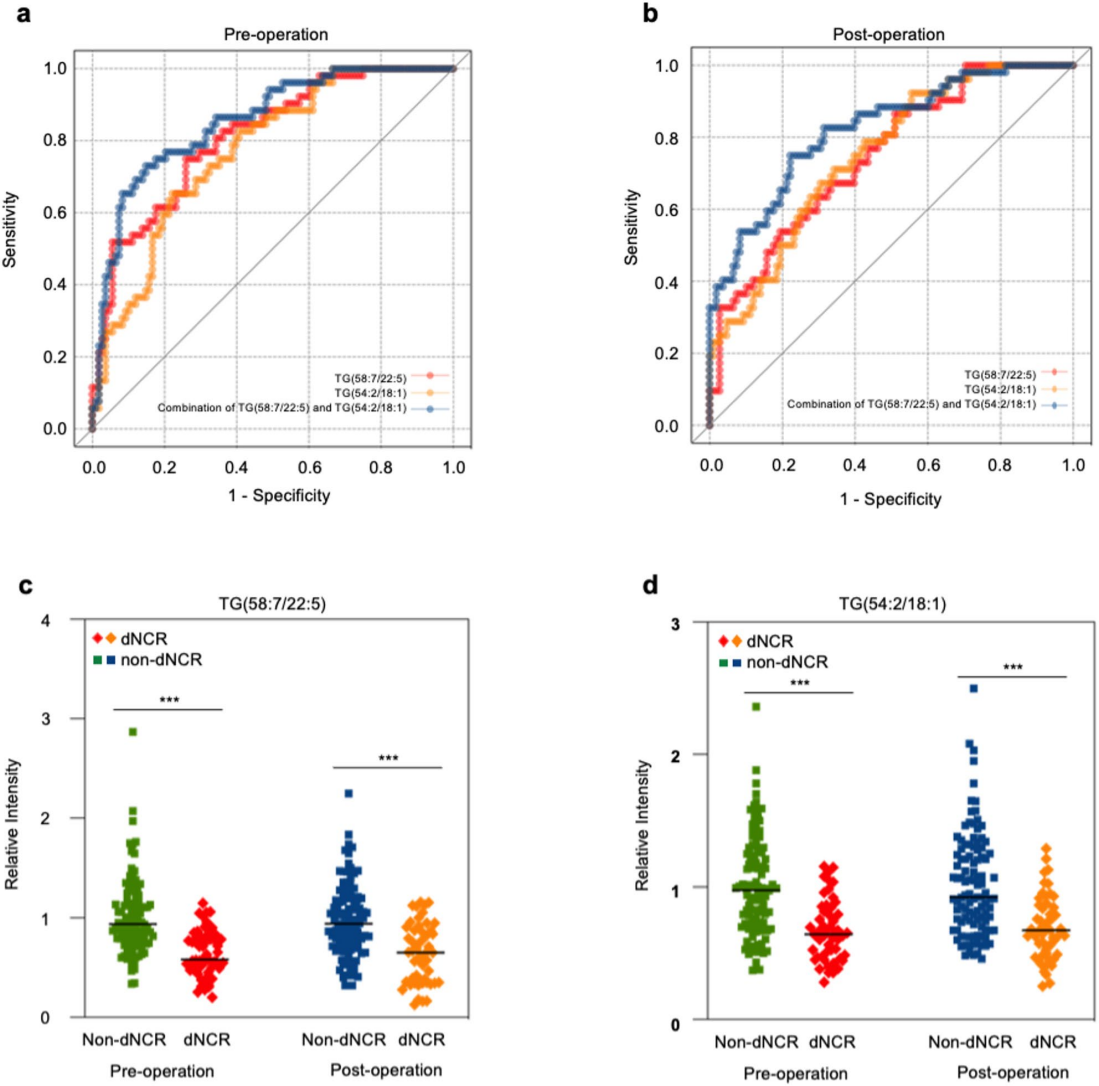
ROC curve for differential lipid metabolites. **a** ROC curve for preoperative lipid metabolites. TG(58:7/22:5), AUC = 0.81, Sensitivity = 0.74, Specificity = 0.75. TG(54:2/18:1), AUC = 0.77, Sensitivity = 0.65, Specificity = 0.78. The combination of TG(58:7/22:5) and TG(54:2/18:1), AUC = 0.86, Sensitivity = 0.73, Specificity = 0.85. **b** ROC curve for postoperative lipid metabolites. TG(58:7/22:5), AUC = 0.75, Sensitivity = 0.87, Specificity = 0.49. TG(54:2/18:1), AUC = 0.75, Sensitivity = 0.71, Specificity = 0.66. The combination of TG(58:7/22:5) and TG(54:2/18:1), AUC = 0.82, Sensitivity = 0.75, Specificity = 0.78. **c** Scatter plots of TG(58:7/22:5) between dNCR and non-dNCR groups. TG(58:7/22:5) was lower in the serum of dNCR group than non-dNCR group before and after surgery (*FDR *< 0.001), and there was no significant change in the serum levels after anaesthesia among all patients. **d** Scatter plots of TG(54:2/18:1) between dNCR and non-dNCR groups. TG(54:2/18:1) was lower in the serum of dNCR group than non-dNCR group before and after surgery (*FDR* < 0.001), and no significant change was found in the serum levels after anaesthesia among patients. Difference in lipid metabolites was analyzed by Student t-test, and *P*-values were adjusted by false discovery rate (*FDR*). *** indicates *FDR* < 0.001. TG = triglyceride

The predictive efficacy of these leading metabolites was subsequently evaluated by ROC analyses (Table 3). Notably, TG(58:7/22:5) demonstrated consistent predictive performance both preoperatively (AUC = 0.81, sensitivity = 0.74, specificity = 0.75; 95% CI 0.74 to 0.88) and postoperatively (AUC = 0.75, sensitivity = 0.87, specificity = 0.49; 95% CI 0.67 to 0.83). Similarly, TG(54:2/18:1) showed reliable diagnostic value preoperatively (AUC = 0.77, sensitivity = 0.65, specificity = 0.78; 95% CI 0.70 to 0.85) and postoperatively (AUC = 0.75, sensitivity = 0.71, specificity = 0.66; 95% CI 0.67 to 0.83) (Fig. 4a, b). ROC analysis was performed on the combination of the above lipid metabolites, revealing that serum TG(58:7/22:5) combined with TG(54:2/18:1) exhibited robust predictive efficiency for dNCR (preoperative AUC = 0.86, sensitivity = 0.73, specificity = 0.85, 95%CI 0.79 to 0.92; postoperative AUC = 0.82, sensitivity = 0.75, specificity = 0.78, 95%CI 0.75 to 0.89) (Fig. 4a, b).

**Table 3:** AUC of preoperative and postoperative differential serum lipid metabolites

Lipid metabolites	AUC	95% CI	Sensitivity	Specificity	*P*-value^*^	Adjusted *P*-value^+^
Preoperative						
TG(58:7/22:5)	0.81	0.74–0.88	0.74	0.75	**< 0.001**	**< 0.001**
TG(54:2/18:1)	0.77	0.70–0.85	0.65	0.78	**< 0.001**	**< 0.001**
TG(58:6/22:5)	0.76	0.68–0.84	0.75	0.66	**< 0.001**	**< 0.001**
TG(58:7/18:1)	0.76	0.68–0.84	0.75	0.66	**< 0.001**	**< 0.001**
TG(58:8/22:5)	0.75	0.68–0.83	0.90	0.47	**< 0.001**	**< 0.001**
TG(54:2/18:0)	0.75	0.67–0.83	0.79	0.61	**< 0.001**	**< 0.001**
Postoperative						
PE(O-16:0/18:1)	0.77	0.69–0.86	0.62	0.90	**< 0.001**	**< 0.001**
TG(56:5/20:4)	0.76	0.68–0.85	0.67	0.79	**< 0.001**	**< 0.001**
TG(54:3/18:1)	0.76	0.68–0.84	0.73	0.70	**< 0.001**	**< 0.001**
TG(56:4/20:4)	0.76	0.68–0.84	0.56	0.88	**< 0.001**	**< 0.001**
TG(54:2/18:1)	0.75	0.67–0.83	0.71	0.66	**< 0.001**	**< 0.001**
TG(58:7/22:5)	0.75	0.67–0.83	0.87	0.49	**< 0.001**	**< 0.001**

*AUC *Area under the curve, *CI *Confidence interval, *TG *Triglyceride, *PE *Phosphatidylethanolamine

* P-values tested the null hypothesis that the AUC equals 0.5. ^+^Adjusted *P*-values were adjusted by the Bonferroni method (n = 10)

The serum levels of TG(58:7/22:5) and TG(54:2/18:1) were significantly lower in the dNCR patients both before and after surgery (*FDR*< 0.001). Importantly, neither metabolite exhibited significant change following anaesthesia in the overall patient cohort (Fig. 4c, d), supporting their stability as potential predictive markers for dNCR.

To ascertain their independent prognostic value, we performed logistic regression analyses for both preoperative and postoperative periods. After adjusted for covariates including SAS and SDS scores, blood loss, and anesthesia duration, TG(58:7/22:5) (preoperative OR = 0.014, 95% CI 0.002–0.109, adjusted *P* < 0.001; postoperative OR = 0.067, 95% CI 0.015–0.309, adjusted *P* = 0.003) and TG(54:2/18:1) (preoperative OR = 0.051, 95% CI 0.010–0.252, adjusted *P* = 0.002; postoperative OR = 0.034, 95% CI 0.006–0.176, adjusted *P* < 0.001) maintained significant associations with dNCR risk in both models, confirming their status as independent risk factors throughout the perioperative period (Table 4).

**Table 4:** Logistic regression of TG(58:7/22:5) and TG(54:2/18:1)

Variables		OR	95% CI	*P*-value	Adjusted *P*-value^*^
Preoperative					
Lipid metabolites	TG(58:7/22:5)	0.014	0.002–0.109	< 0.001	**< 0.001**
	TG(54:2/18:1)	0.051	0.010–0.252	< 0.001	**0.002**
Covariates	SAS	0.875	0.768–0.997	0.044	0.264
	SDS	1.031	0.915–1.162	0.611	> 0.999
	Bleeding volume	1.000	0.997–1.003	0.988	> 0.999
	Anesthesia duration	0.999	0.995–1.003	0.607	> 0.999
Postoperative					
Lipid metabolites	TG(58:7/22:5)	0.067	0.015–0.309	< 0.001	**0.003**
	TG(54:2/18:1)	0.034	0.006–0.176	< 0.001	**< 0.001**
Covariates	SAS	0.880	0.770–1.005	0.059	0.354
	SDS	0.992	0.880–1.119	0.902	> 0.999
	Bleeding volume	1.000	0.996–1.003	0.794	> 0.999
	Anesthesia duration	0.999	0.995–1.004	0.798	> 0.999

*OR *Odds ratio, *CI *Confidence interval, *TG *Triglyceride, *SAS *Self-Rating Anxiety Scale, *SDS *Self-Rating Depression Scale

^*^The *P*-values from the logistic regression analyses were adjusted by Bonferroni correction with a factor of n = 6

## Discussion

Lipids are fundamental constituents of neuronal membranes, essential for myelin synthesis, synaptic plasticity, and energy metabolism [26]. Inhaled anesthetics disrupt lipid rafts, which specialize in membrane microdomains crucial for cellular signaling and protein sorting [27, 28]. Previous studies proved that chronic sevoflurane exposure induced prefrontal cortex lipid remodeling in aged marmosets and mice [29]. However, human neural lipid metabolism under anaesthesia is still under studies. By employing widely-targeted metabolomics, this study identifies two serum TG species, TG(58:7/22:5) and TG(54:2/18:1), as novel biomarkers for dNCR in geriatric patients undergoing oral-maxillofacial surgery. These results highlight perioperative lipidomic profiling as a promising tool for early cognitive risk assessment and underscore potential abnormalities in TG metabolism associated with dNCR.

TGs are glycerol esters linked with three fatty acids and serve as critical energy reservoir in the brain, directly supporting synaptic function. According to the study by Kumar et al., TGs store in astrocytes can be rapidly broken down when glucose supply is insufficient, providing on-demand fuel for synaptic activity through β-oxidation. Notably, neurons themselves are also capable of utilizing intracellular TG reserves, mobilizing these energy stores during metabolic stress to sustain synaptic function [30]. This insight offers a new perspective for understanding perioperative neurocognitive disorders. Surgical stress may lead to excessive depletion of brain TG reserves, and if patients already have pre-existing deficiencies in specific TG species, this may result in impaired synaptic energy supply and subsequent cognitive dysfunction.

Recent clinical evidence suggests an age-dependent neuroprotective effect of TGs [31, 32]. Higher circulating TG levels are associated with slower cognitive decline and reduced dementia risk in older population [33]. Specific TG species serve as carriers of long-chain fatty acids like docosapentaenoic acid (DPA) and oleic acid. Upon hydrolysis, these fatty acids become available for maintaining neuronal membrane integrity and synaptic function. Reduced availability of these fatty acids could impair neuronal energy homeostasis, synaptic plasticity, and neuroprotection [34]. Indeed, TG species containing long-chain polyunsaturated fatty acids are depleted in Alzheimer’s disease (AD) and mild cognitive impairment. Lower plasma oleic acid levels have been linked to increased AD risk, whereas higher serum omega-3 DPA concentrations correlate positively with cognitive performance in older adults [35]. These observations suggest chronically low serum TG levels may reflect deficits in these protective fatty acids, contributing to cognitive vulnerability.

Previous studies in cardiac surgery have identified postoperative phosphatidylinositol, sphingomyelin, sphingolipids, glycerophospholipids, and fatty acids as correlates of dNCR [14, 21]. Our work uniquely establishes perioperative TGs as predictive biomarkers. This underscores the importance of preexisting metabolic state in postoperative cognitive outcomes, beyond acute surgical stress responses.

The observed TG alterations align with evidence from Alzheimer’s disease biomarker studies, further implicating overlapping pathways in perioperative and chronic neurodegeneration such as membrane destabilization, synaptic vulnerability, and amyloid processing. Further in-depth analysis is needed to clarify the function of specific lipid metabolites in both dNCR and neurodegenerative disease.

Altered levels of specific TGs and fatty acids were observed in studies of Alzheimer’s disease and mild cognitive impairment research. However, Shen et al. reported that preoperative hyperlipidemia, especially elevated cholesterol, was an independent risk factor for postoperative cognitive dysfunction [36]. Similarly, higher preoperative triglyceride level was associated with increased delirium risk after colorectal cancer surgery [37]. Additionally, Parthasarathy et al. found that higher serum triglycerides predicted worse executive function in non-demented elderly [38]. These findings may seem inconsistent with ours. The discrepancy could be attributed to differences in surgical procedures, patient populations, or the absence of detailed lipid molecular subtyping. These studies reveal a complex, age-dependent relationship between lipids and cognition and collectively emphasize the importance of distinguishing specific lipid species rather than focusing solely on overall lipid levels.

Despite lipid metabolism disorders, abnormal glucose metabolism may also contribute to postoperative cognitive dysfunction. Clinical study has confirmed that serum levels of glucose metabolites (oxaloacetate and 2-aminoadipic acid) are significantly associated with dNCR [39]. Furthermore, the triglyceride-glucose index has been proved as predictive value for cognitive impairment in elderly patients [40, 41]. Future studies could explore integrated metabolic biomarker models to enhance dNCR prediction.

Clinically, our results offer valuable insights for perioperative management. The predictive capability of TG(58:7/22:5) and TG(54:2/18:1) (AUC = 0.86 ) suggests their potential inclusion in preoperative cognitive risk stratification. Elderly patients with significantly low serum TG levels could be identified for closer perioperative cognitive monitoring or tailored interventions. Also, lipid profiling might complement existing clinical assessments, enabling a more comprehensive evaluation of cognitive vulnerability.

In addition, higher preoperative SAS and SDS scores were found in the non-dNCR group in this study, which could be mainly explained by the cognitive reserve hypothesis. Preoperative psychological distress may reflect enhanced neural reactivity that inadvertently potentiates cognitive resilience through improved neural plasticity [42]. When confronted with surgical stress, these patients demonstrate superior capacity to recruit compensatory neural networks and maintain metabolic homeostasis. Furthermore, clinically evident anxiety likely prompted more intensive perioperative management—including optimized analgesia and psychological support—creating an additional protective effect. Furthermore, the self-reported nature of the SAS and SDS scales requires adequate attentional capacity and self-awareness, which may be compromised in patients with pre-existing mild cognitive frailty or those who develop postoperative dNCR. As a result, such individuals might underreport or inaccurately represent their emotional symptoms, potentially leading to artificially low scores on these measures.

This study has several limitations. First, standardized neurocognitive assessments were performed after hospital admission, which may lead to potential bias induced by hospitalization-related stress. Second, the absence of a multivariate prediction model integrating clinical and lipidomic variables limits the personalized risk stratification. Third, the restricted sampling timepoints may not fully capture dynamic metabolic changes throughout the perioperative period. Finally, the exclusion of patients with POD constrains the generalizability of our findings to complex populations with comorbid POD.

## Conclusions

This study employed widely-targeted metabolomics to characterize perioperative serum lipid profiles in geriatric patients undergoing oral and maxillofacial surgeries. We established specific serum TG species as novel biomarkers for dNCR in elderly surgical patients, underscoring lipid metabolism as a potentially modifiable factor in perioperative cognitive care.

## Data Availability

The datasets analysed during the current study are available from the corresponding author on reasonable request.

## Abbreviations

PND: Perioperative neurocognitive disorders
dNCR: Delayed neurocognitive recovery
POD: Postoperative delirium
BBB: Blood-brain barrier
ASA: American Society of Anesthesiologists
SD: Standard deviation
DSM-5: Diagnostic and Statistical Manual of Mental Disorders Fifth Edition
MMSE: Mini-Mental State Examination
MoCA: Montreal Cognitive Assessment
SDS: Self-Rating Depression Scale
SAS: Self-Rating Anxiety Scale
BIS: Bispectral index
LC-MS: Liquid chromatography–mass spectrometry
OPLS-DA: Orthogonal partial least squares discriminant analysis
VIP: Variable importance in projection
FC: Fold-change
FDR: False discovery rate
ROC: Receiver operating characteristic
AUC: Area under the curve
TG: Triglyceride
FA: Fatty acid
PE: Phosphatidylethanolamine
CL: Cardiolipin
DPA: Docosapentaenoic acid
AD: Alzheimer’s disease
